# Cardiovascular activity in migraine patients: Influence of age and headache chronification

**DOI:** 10.1186/1129-2377-14-S1-P139

**Published:** 2013-02-21

**Authors:** O Grosu, S Odobescu, I Moldovanu, L Rotaru

**Affiliations:** 1 Institute of Neurology and Neurosurgery, Moldova, Republic of

## Introduction

Heart rate variability (HRV) is a useful tool to evaluate cardiovascular activity in different pathologies including migraine. Reduced HRV over 24 hours predicts increase in cardio-vascular morbidity.

## Objectives

To evaluate and analyze cardiovascular activity by mean of HRV in migraine patients and elucidate the influence of age and chronification.

## Methods

Study group consisted of 65 pts with migraine: 40 pts with chronic (CM) and 25 pts with episodic migraine (EM), mean age 47.7±11.29 (CM) and 47.6±12.6 years (EM). All the patients underwent 24-hour ambulatory ECG monitoring with evaluation of HRV. The analyzed time domain parameters were: VAR, CBBP, avNN, SDNN, RRNN, SDANN, SDNN index, RMSSD and pNN50 % and frequency domain parameters: LF, HF and VLF. All expressed as mean, day time and night time values.

## Results

CM patients had increased VAR (946.8±433.4 vs. 785.6±214.4, p<0.05), SDNN index (53,1±17.8 vs. 45.7±8.6, p<0.05) and VLF (2013.05±1151.5 vs. 1567.32±526.6, p<0.05). In the middle age (40-50 years old) EM patients had increased Heart rate (84.8±8.6 vs. 77.5± 7.9, p<0.05) and CM patients increased: avNN (837.6±93.7 vs. 787.0±78.3, p<0.05), SDNN index (60.1±15.0 vs. 45.8±5.63, p<0.05) and VLF (2724.4±1449.1 vs. 1618.2±509.9, p<0.05). In the advanced age (50-59 years old) CM patients had increased HF (30.6±13.1 vs. 19.7±11.9, p<0.05). Compared between groups (EM40-50 vs. EM 50-59) - EM50-59 patients had reduced pNN50% and CBBP and CM 50-59 patients had reduced pNN50%, SDNN index, VLF, LF, HF but increased HF(%). Conclusions CM patients presented an increased HRV and parasympathetic activity compared with EM in whole study sample and in the 40-50 age group but just parasympathetic hyperactivity in the 50-59 age groups. Older CM patients presented more reduction in total HRV, sympathetic and parasympathetic influence (day time), except HF (%) night time which reflect strong vagal influence probably as a consequence of chronification process.
